# Supplementary material for: HIVprotI: an integrated web based platform for prediction and design of HIV proteins inhibitors
Source: J Cheminform. 2018 Mar 9;10:12. doi: 10.1186/s13321-018-0266-y (PMC5845081; doi:10.1186/s13321-018-0266-y)
Supplement: Supplementary file 2 — Additional file 2. Source code of HIVProtI web server. [file 13321_2018_266_MOESM2_ESM.zip › HIVprotI_Source-code/hivprotI/weka-3-6-6/doc/weka/gui/treevisualizer/Colors.html]

Colors


---


|  |  |  |
| --- | --- | --- |
| **Overview** \| **Package** \| **Class** \| **Tree** \| **Deprecated** \| **Index** \| **Help** \| **Weka's home** | |  |
| PREV CLASS   **NEXT CLASS** | **FRAMES**    **NO FRAMES**     **All Classes** |  |
| SUMMARY: NESTED \| FIELD \| CONSTR \| METHOD | DETAIL: FIELD \| CONSTR \| METHOD |  |


---


## weka.gui.treevisualizer Class Colors

```
java.lang.Object
  weka.gui.treevisualizer.Colors
```

---

``` public class Colors extends java.lang.Object ```

This class maintains a list that contains all the colornames from the
dotty standard and what color (in RGB) they represent

**Version:**
:   $Revision: 1.5 $

**Author:**
:   Malcolm Ware (mfw4@cs.waikato.ac.nz)

---

| **Field Summary** | |
| --- | --- |
| `NamedColor[]` | `m_cols`             The array with all the colors input |


**Constructor Summary**

`Colors()`


**Method Summary**

**Methods inherited from class java.lang.Object**

`equals, getClass, hashCode, notify, notifyAll, toString, wait, wait, wait`

**Field Detail**

### m\_cols

```
public NamedColor[] m_cols
```

:   The array with all the colors input


**Constructor Detail**

### Colors

```
public Colors()
```


---


|  |  |  |
| --- | --- | --- |
| **Overview** \| **Package** \| **Class** \| **Tree** \| **Deprecated** \| **Index** \| **Help** \| **Weka's home** | |  |
| PREV CLASS   **NEXT CLASS** | **FRAMES**    **NO FRAMES**     **All Classes** |  |
| SUMMARY: NESTED \| FIELD \| CONSTR \| METHOD | DETAIL: FIELD \| CONSTR \| METHOD |  |


---
